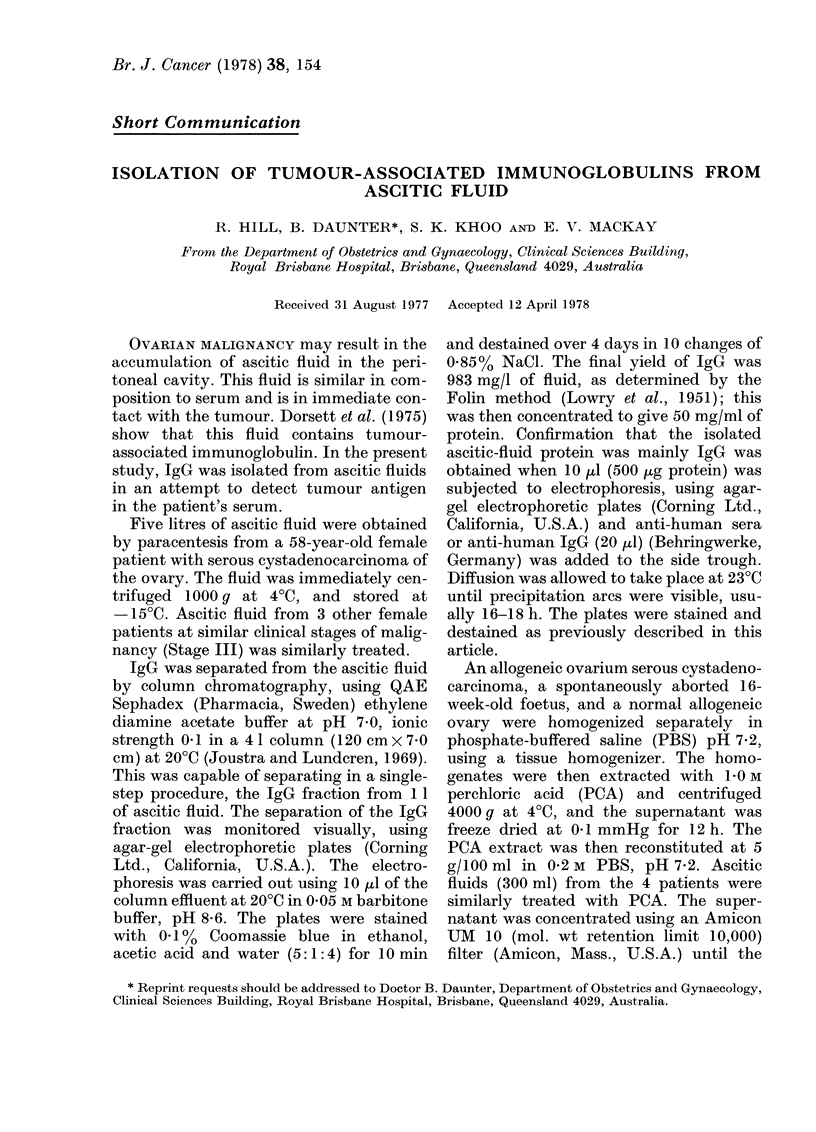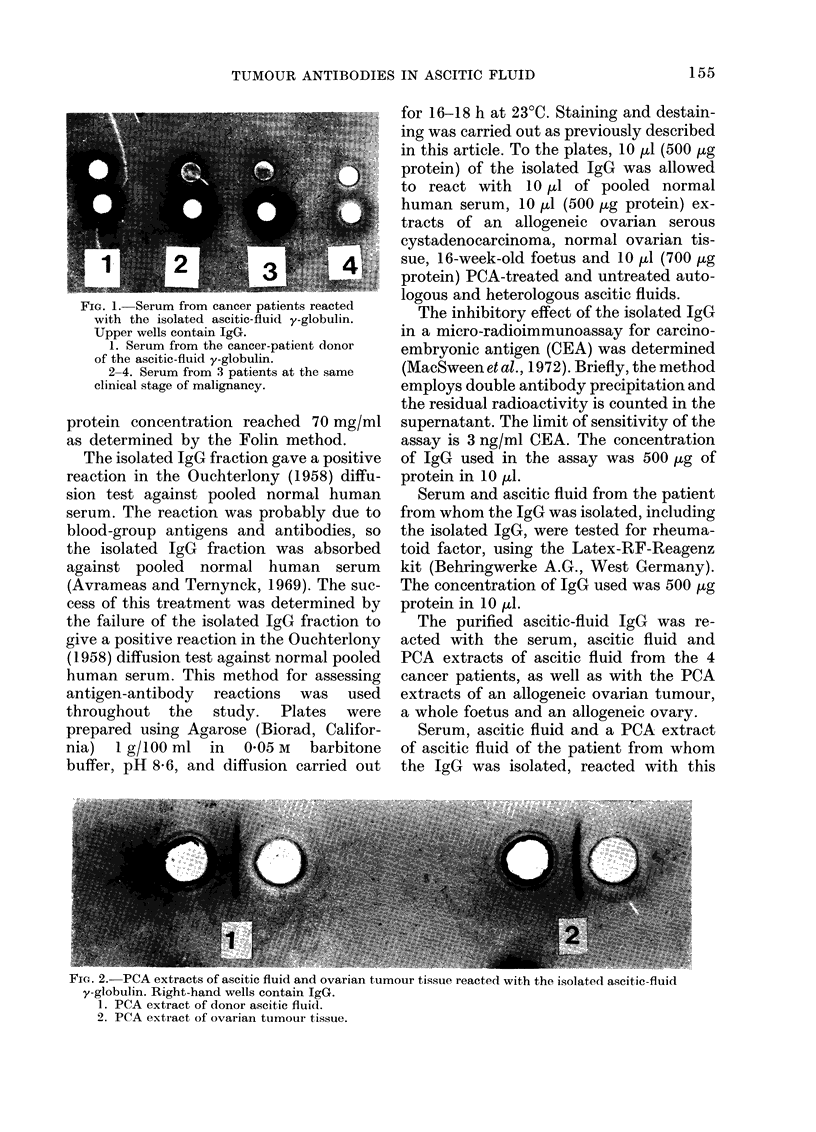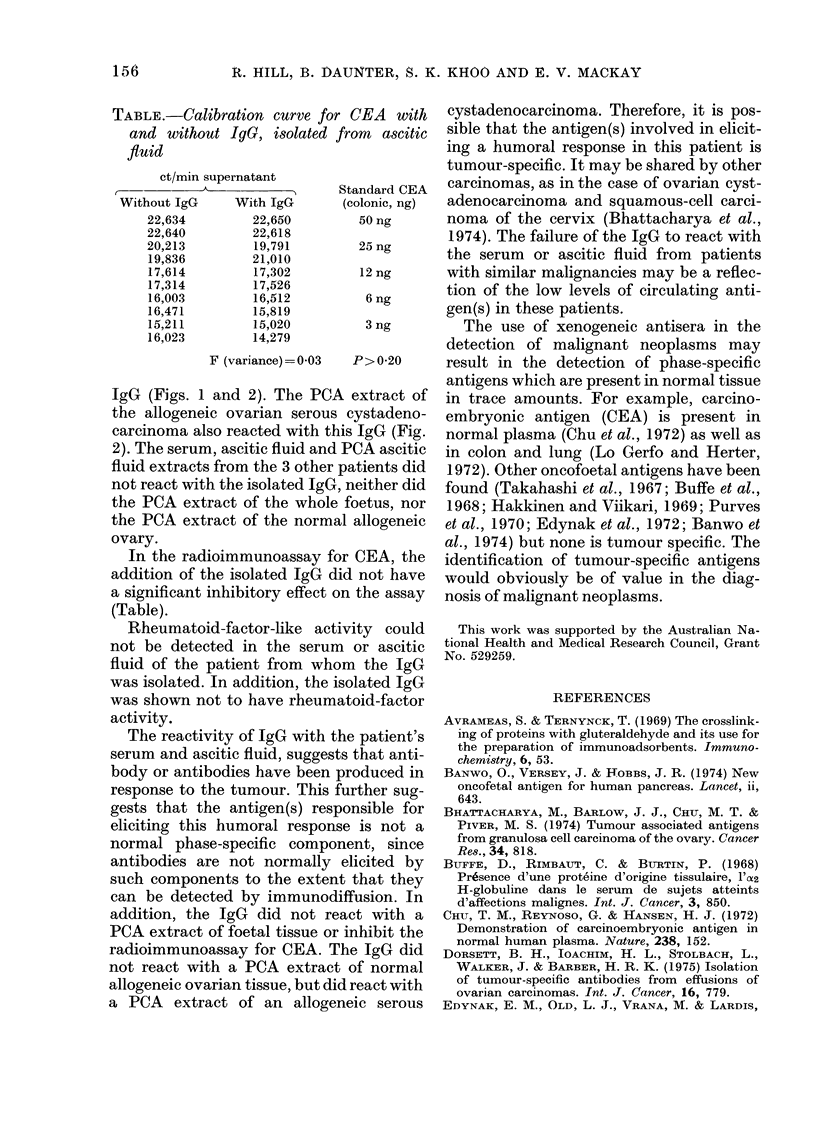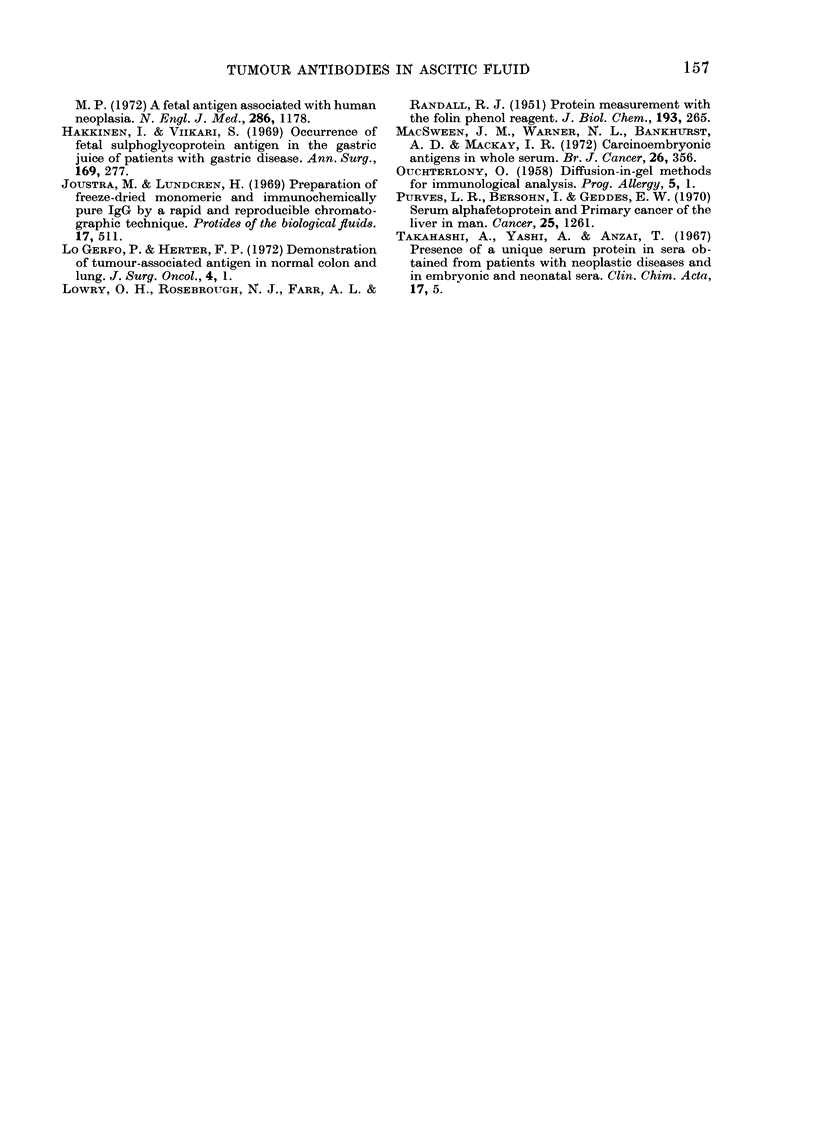# Isolation of tumour-associated immunoglobulins from ascitic fluid.

**DOI:** 10.1038/bjc.1978.177

**Published:** 1978-07

**Authors:** R. Hill, B. Daunter, S. K. Khoo, E. V. MacKay

## Abstract

**Images:**


*Br. J. Cancer* (1978) **38**, 154

***Short Communication***

### ISOLATION OF TUMOUR-ASSOCIATED IMMUNOGLOBULINS FROM ASCITIC FLUID

R. HILL, B. DAUNTER*, S. K. KHOO AND E. V. MACKAY

*From the Department of Obstetrics and Gynaecology, Clinical Sciences Building, Royal Brisbane Hospital, Brisbane, Queensland* 4029, *Australia*

Received 31 August 1977 Accepted 12 April 1978

OVARIAN MALIGNANCY may result in the accumulation of ascitic fluid in the peritoneal cavity. This fluid is similar in composition to serum and is in immediate contact with the tumour. Dorsett *et al.* (1975) show that this fluid contains tumour-associated immunoglobulin. In the present study, IgG was isolated from ascitic fluids in an attempt to detect tumour antigen in the patient's serum.

Five litres of ascitic fluid were obtained by paracentesis from a 58-year-old female patient with serous cystadenocarcinoma of the ovary. The fluid was immediately centrifuged 1000 *g* at 4°C, and stored at −15°C. Ascitic fluid from 3 other female patients at similar clinical stages of malignancy (Stage III) was similarly treated.

IgG was separated from the ascitic fluid by column chromatography, using QAE Sephadex (Pharmacia, Sweden) ethylene diamine acetate buffer at pH 7·0, ionic strength 0·1 in a 4 l column (120 cm × 7·0 cm) at 20°C (Joustra and Lundcren, 1969). This was capable of separating in a single-step procedure, the IgG fraction from 1 l of ascitic fluid. The separation of the IgG fraction was monitored visually, using agar-gel electrophoretic plates (Corning Ltd., California, U.S.A.). The electrophoresis was carried out using 10 μl of the column effluent at 20°C in 0·05 M barbitone buffer, pH 8·6. The plates were stained with 0·1% Coomassie blue in ethanol, acetic acid and water (5:1:4) for 10 min and destained over 4 days in 10 changes of 0·85% NaCl. The final yield of IgG was 983 mg/l of fluid, as determined by the Folin method (Lowry *et al.*, 1951); this was then concentrated to give 50 mg/ml of protein. Confirmation that the isolated ascitic-fluid protein was mainly IgG was obtained when 10 μl (500 μg protein) was subjected to electrophoresis, using agar-gel electrophoretic plates (Corning Ltd., California, U.S.A.) and anti-human sera or anti-human IgG (20 μl) (Behringwerke, Germany) was added to the side trough. Diffusion was allowed to take place at 23°C until precipitation arcs were visible, usually 16–18 h. The plates were stained and destained as previously described in this article.

An allogeneic ovarium serous cystadenocarcinoma, a spontaneously aborted 16-week-old foetus, and a normal allogeneic ovary were homogenized separately in phosphate-buffered saline (PBS) pH 7·2, using a tissue homogenizer. The homogenates were then extracted with 1·0 M perchloric acid (PCA) and centrifuged 4000 *g* at 4°C, and the supernatant was freeze dried at 0·1 mmHg for 12 h. The PCA extract was then reconstituted at 5 g/100 ml in 0·2 M PBS, pH 7·2. Ascitic fluids (300 ml) from the 4 patients were similarly treated with PCA. The supernatant was concentrated using an Amicon UM 10 (mol. wt retention limit 10,000) filter (Amicon, Mass., U.S.A.) until the

* Reprint requests should be addressed to Doctor B. Daunter, Department of Obstetrics and Gynaecology, Clinical Sciences Building, Royal Brisbane Hospital, Brisbane, Queensland 4029, Australia.

FIG. 1.—Serum from cancer patients reacted with the isolated ascitic-fluid $\gamma$-globulin. Upper wells contain IgG.

1. Serum from the cancer-patient donor of the ascitic-fluid $\gamma$-globulin.

2–4. Serum from 3 patients at the same clinical stage of malignancy.

protein concentration reached 70 mg/ml as determined by the Folin method.

The isolated IgG fraction gave a positive reaction in the Ouchterlony (1958) diffusion test against pooled normal human serum. The reaction was probably due to blood-group antigens and antibodies, so the isolated IgG fraction was absorbed against pooled normal human serum (Avrameas and Ternynck, 1969). The success of this treatment was determined by the failure of the isolated IgG fraction to give a positive reaction in the Ouchterlony (1958) diffusion test against normal pooled human serum. This method for assessing antigen-antibody reactions was used throughout the study. Plates were prepared using Agarose (Biorad, California) 1 g/100 ml in 0·05 M barbitone buffer, pH 8·6, and diffusion carried out for 16–18 h at 23°C. Staining and destaining was carried out as previously described in this article. To the plates, 10 $\mu$l (500 $\mu$g protein) of the isolated IgG was allowed to react with 10 $\mu$l of pooled normal human serum, 10 $\mu$l (500 $\mu$g protein) extracts of an allogeneic ovarian serous cystadenocarcinoma, normal ovarian tissue, 16-week-old foetus and 10 $\mu$l (700 $\mu$g protein) PCA-treated and untreated autologous and heterologous ascitic fluids.

The inhibitory effect of the isolated IgG in a micro-radioimmunoassay for carcinoembryonic antigen (CEA) was determined (MacSween *et al.*, 1972). Briefly, the method employs double antibody precipitation and the residual radioactivity is counted in the supernatant. The limit of sensitivity of the assay is 3 ng/ml CEA. The concentration of IgG used in the assay was 500 $\mu$g of protein in 10 $\mu$l.

Serum and ascitic fluid from the patient from whom the IgG was isolated, including the isolated IgG, were tested for rheumatoid factor, using the Latex-RF-Reagenz kit (Behringwerke A.G., West Germany). The concentration of IgG used was 500 $\mu$g protein in 10 $\mu$l.

The purified ascitic-fluid IgG was reacted with the serum, ascitic fluid and PCA extracts of ascitic fluid from the 4 cancer patients, as well as with the PCA extracts of an allogeneic ovarian tumour, a whole foetus and an allogeneic ovary.

Serum, ascitic fluid and a PCA extract of ascitic fluid of the patient from whom the IgG was isolated, reacted with this

FIG. 2.—PCA extracts of ascitic fluid and ovarian tumour tissue reacted with the isolated ascitic-fluid $\gamma$-globulin. Right-hand wells contain IgG.

1. PCA extract of donor ascitic fluid.
2. PCA extract of ovarian tumour tissue.

TABLE.—*Calibration curve for CEA with and without IgG, isolated from ascitic fluid*

| ct/min supernatant | | Standard CEA |
|---|---|---|
| Without IgG | With IgG | (colonic, ng) |
| 22,634 | 22,650 | 50 ng |
| 22,640 | 22,618 | |
| 20,213 | 19,791 | 25 ng |
| 19,836 | 21,010 | |
| 17,614 | 17,302 | 12 ng |
| 17,314 | 17,526 | |
| 16,003 | 16,512 | 6 ng |
| 16,471 | 15,819 | |
| 15,211 | 15,020 | 3 ng |
| 16,023 | 14,279 | |
| | F (variance) = 0·03 | $P > 0 \cdot 20$ |

IgG (Figs. 1 and 2). The PCA extract of the allogeneic ovarian serous cystadenocarcinoma also reacted with this IgG (Fig. 2). The serum, ascitic fluid and PCA ascitic fluid extracts from the 3 other patients did not react with the isolated IgG, neither did the PCA extract of the whole foetus, nor the PCA extract of the normal allogeneic ovary.

In the radioimmunoassay for CEA, the addition of the isolated IgG did not have a significant inhibitory effect on the assay (Table).

Rheumatoid-factor-like activity could not be detected in the serum or ascitic fluid of the patient from whom the IgG was isolated. In addition, the isolated IgG was shown not to have rheumatoid-factor activity.

The reactivity of IgG with the patient's serum and ascitic fluid, suggests that antibody or antibodies have been produced in response to the tumour. This further suggests that the antigen(s) responsible for eliciting this humoral response is not a normal phase-specific component, since antibodies are not normally elicited by such components to the extent that they can be detected by immunodiffusion. In addition, the IgG did not react with a PCA extract of foetal tissue or inhibit the radioimmunoassay for CEA. The IgG did not react with a PCA extract of normal allogeneic ovarian tissue, but did react with a PCA extract of an allogeneic serous cystadenocarcinoma. Therefore, it is possible that the antigen(s) involved in eliciting a humoral response in this patient is tumour-specific. It may be shared by other carcinomas, as in the case of ovarian cystadenocarcinoma and squamous-cell carcinoma of the cervix (Bhattacharya *et al.*, 1974). The failure of the IgG to react with the serum or ascitic fluid from patients with similar malignancies may be a reflection of the low levels of circulating antigen(s) in these patients.

The use of xenogeneic antisera in the detection of malignant neoplasms may result in the detection of phase-specific antigens which are present in normal tissue in trace amounts. For example, carcinoembryonic antigen (CEA) is present in normal plasma (Chu *et al.*, 1972) as well as in colon and lung (Lo Gerfo and Herter, 1972). Other oncofoetal antigens have been found (Takahashi *et al.*, 1967; Buffe *et al.*, 1968; Hakkinen and Viikari, 1969; Purves *et al.*, 1970; Edynak *et al.*, 1972; Banwo *et al.*, 1974) but none is tumour specific. The identification of tumour-specific antigens would obviously be of value in the diagnosis of malignant neoplasms.

This work was supported by the Australian National Health and Medical Research Council, Grant No. 529259.

### REFERENCES

AVRAMEAS, S. & TERNYNCK, T. (1969) The crosslinking of proteins with gluteraldehyde and its use for the preparation of immunoadsorbents. *Immunochemistry*, **6**, 53.

BANWO, O., VERSEY, J. & HOBBS, J. R. (1974) New oncofetal antigen for human pancreas. *Lancet*, ii, 643.

BHATTACHARYA, M., BARLOW, J. J., CHU, M. T. & PIVER, M. S. (1974) Tumour associated antigens from granulosa cell carcinoma of the ovary. *Cancer Res.*, **34**, 818.

BUFFE, D., RIMBAUT, C. & BURTIN, P. (1968) Présence d'une protéine d'origine tissulaire, l'$\alpha_2$ H-globuline dans le serum de sujets atteints d'affections malignes. *Int. J. Cancer*, **3**, 850.

CHU, T. M., REYNOSO, G. & HANSEN, H. J. (1972) Demonstration of carcinoembryonic antigen in normal human plasma. *Nature*, **238**, 152.

DORSETT, B. H., IOACHIM, H. L., STOLBACH, L., WALKER, J. & BARBER, H. R. K. (1975) Isolation of tumour-specific antibodies from effusions of ovarian carcinomas. *Int. J. Cancer*, **16**, 779.

EDYNAK, E. M., OLD, L. J., VRANA, M. & LARDIS,

M. P. (1972) A fetal antigen associated with human neoplasia. *N. Engl. J. Med.*, **286**, 1178.

HAKKINEN, I. & VIIKARI, S. (1969) Occurrence of fetal sulphoglycoprotein antigen in the gastric juice of patients with gastric disease. *Ann. Surg.*, **169**, 277.

JOUSTRA, M. & LUNDCREN, H. (1969) Preparation of freeze-dried monomeric and immunochemically pure IgG by a rapid and reproducible chromatographic technique. *Protides of the biological fluids.* **17**, 511.

LO GERFO, P. & HERTER, F. P. (1972) Demonstration of tumour-associated antigen in normal colon and lung. *J. Surg. Oncol.*, **4**, 1.

LOWRY, O. H., ROSEBROUGH, N. J., FARR, A. L. & RANDALL, R. J. (1951) Protein measurement with the folin phenol reagent. *J. Biol. Chem.*, **193**, 265.

MACSWEEN, J. M., WARNER, N. L., BANKHURST, A. D. & MACKAY, I. R. (1972) Carcinoembryonic antigens in whole serum. *Br. J. Cancer*, **26**, 356.

OUCHTERLONY, O. (1958) Diffusion-in-gel methods for immunological analysis. *Prog. Allergy*, **5**, 1.

PURVES, L. R., BERSOHN, I. & GEDDES, E. W. (1970) Serum alphafetoprotein and Primary cancer of the liver in man. *Cancer*, **25**, 1261.

TAKAHASHI, A., YASHI, A. & ANZAI, T. (1967) Presence of a unique serum protein in sera obtained from patients with neoplastic diseases and in embryonic and neonatal sera. *Clin. Chim. Acta*, **17**, 5.